# Functional Imaging of the Outer Retinal Complex using High Fidelity Imaging Retinal Densitometry

**DOI:** 10.1038/s41598-020-60660-9

**Published:** 2020-03-11

**Authors:** Tom H. Margrain, David Atkinson, Alison M. Binns, James Fergusson, Allannah Gaffney, David Henry, Chris Jones, Trevor D. Lamb, Dave Melotte, Chris Miller, Stephen Todd, Ashley Wood

**Affiliations:** 1 School of Optometry and Vision Sciences, Maindy Road, Cardiff University, Cardiff, Wales CF24 4HQ UK; 2 UK Astronomy Technology Centre, Royal Observatory, Edinburgh, Blackford Hill, Edinburgh, EH9 3HJ UK; 3 School of Health Sciences, City, University of London, Northampton Square, London, EC1V 0HB UK; 4 Eccles Institute of Neuroscience, John Curtin School of Medical Research, The Australian National University, Canberra, ACT 2601 Australia

**Keywords:** Optical imaging, Macular degeneration, Translational research

## Abstract

We describe a new technique, high fidelity Imaging Retinal Densitometry (IRD), which probes the functional integrity of the outer retinal complex. We demonstrate the ability of the technique to map visual pigment optical density and synthesis rates in eyes with and without macular disease. A multispectral retinal imaging device obtained precise measurements of retinal reflectance over space and time. Data obtained from healthy controls and 5 patients with intermediate AMD, before and after photopigment bleaching, were used to quantify visual pigment metrics. Heat maps were plotted to summarise the topography of rod and cone pigment kinetics and descriptive statistics conducted to highlight differences between those with and without AMD. Rod and cone visual pigment synthesis rates in those with AMD (*v* = 0.043 SD 0.019 min^−1^ and *v* = 0.119 SD 0.046 min^−1^, respectively) were approximately half those observed in healthy controls (*v* = 0.079 SD 0.024 min^−1^ for rods and v = 0.206 SD 0.069 min^−1^ for cones). By mapping visual pigment kinetics across the central retina, high fidelity IRD provides a unique insight into outer retinal complex function. This new technique will improve the phenotypic characterisation, diagnosis and treatment monitoring of various ocular pathologies, including AMD.

## Introduction

Continuous photoreceptor function is dependent on the constant renewal of visual pigment molecules by a physiological process known as the visual cycle^1^. Remarkably, the key enzymatic steps in the canonical visual cycle reside not in the photoreceptors but in the cells that nourish them, the retinal pigment epithelium (RPE). More specifically, the RPE converts the retinoid all-*trans* retinol back to 11-*cis* retinal via a series of relatively slow enzymatic reactions which, in the healthy eye, determine the rate at which visual pigment molecules can regenerate^2^. Hence by measuring rod photoreceptor visual pigment synthesis rates we can probe a key aspect of RPE physiology. The RPE also supports the regeneration of visual pigments in cone photoreceptors but an additional visual cycle pathway that involves the neural retina’s Müller Cells is also involved^2,3^.

The RPE, Bruch’s membrane and the choriocapillaris, are associated with many ocular pathologies, most notably age-related macular degeneration (AMD) the developed world’s leading cause of sight loss^4,5^. In AMD, an increase in the thickness of basal laminar deposits, the presence of soft drusen and choriocapillaris drop out all work to impair normal RPE function^5^.

Hence, by measuring visual pigment synthesis rates we not only obtain information about the status of a vital physiological process, the visual cycle, but also information about the functional integrity of the outer retinal complex i.e. the photoreceptors, RPE, Müller Cells, Bruch’s membrane and the choriocapillaris.

Fortunately, visual pigment synthesis rates can be measured by recording the subtle changes in retinal reflectance associated with the visual cycle using a technique called densitometry^6,7^. Although the technique of retinal densitometry was pioneered in the 1950s, the full potential of the technique, to deliver functional imaging of the retina *in vivo*, has not yet been realised because of persistent and intractable technological challenges associated with precise retinal reflection measurements over space and time^7^. Previous retinal densitometry techniques have provided information from one point on the retina only^6,8–13^, limited spectral information^6,8,14,15^, or no temporal information^9,16,17^.

One of the major challenges associated with imaging retinal densitometry (IRD) has been maintaining precise optical alignment with the eye over a period of several minutes, during visual pigment synthesis. The traditional solution to the problem of optical alignment, using a dental impression, is not practical in a clinical setting. An alternative approach is to use active optics systems which have been used extensively in the field of astronomy. In these systems one or more optical elements are actively controlled to compensate for misalignments of a telescope’s opto-mechanical assembly. We reasoned that a similar approach could be used to maintain precise alignment with the pupil of the eye in three dimensions without the need for a dental impression.

An additional challenge in retinal densitometry has been backscatter from the eye’s optical media anterior to the visual pigments. This scattered light dilutes the signal from the visual pigments, leading to a wavelength dependent attenuation of the optical density signal recorded, and causing an apparent spectral shift toward longer wavelengths^18^. Scanning laser ophthalmoscopes minimise scattered light but the limited number of wavelengths available makes teasing apart reflectance changes attributable to different visual pigments challenging^19^. Wide-field imaging scanning laser ophthalmoscopes have been used to probe rod visual pigment kinetics by studying reflectance changes at peripheral retinal locations where rods are in abundance^15^ and adaptive optics systems have been used to study pigment kinetics at the level of individual photoreceptors^14^. Whilst this is advantageous in as much as reflectance changes arising from rods and cones can be differentiated, the signal to noise ratio is so low that cone only data are unreliable, and significant averaging is needed to recover a reliable rod signal^14^. Further complexities are introduced by the presence of transient photo-intermediates, such as metarhodopsin III. These factors cause distortions in the signal and are a barrier to functional imaging because the rate at which visual pigment molecules regenerate cannot be measured with precision.

Here we combine multispectral retinal imaging, 3D infra-red (IR) eye tracking, actively controlled reflective relay optics and back scatter correction to unlock the potential of imaging retinal densitometry (IRD) and deliver functional imaging of the outer retinal complex in 4 dimensions (x, y, λ, t).

The overarching aim of this study was to validate this novel functional imaging modality and to highlight its diagnostic potential in a small group of people with age-related macular degeneration. We map the topographical distribution of rod and cone visual pigments in healthy controls and confirm rate-limited visual pigment kinetics in cones and also in rods after correction for metarhodopsin III. We draw attention to previously unknown topographical variations in absolute pigment regeneration rates and highlight the diagnostic potential of this technique by probing retinal function in a small group of people with and without AMD.

## Methods

Following a laboratory study to benchmark the technology, a prospective observational study was used to measure rod and cone visual pigment metrics in people with and without retinal disease. The study took place at the Cardiff Centre for Vision Sciences and volunteers provided their written informed consent prior to any investigations. All procedures adhered to the Declaration of Helsinki, and ethical approval was provided by the institutional review board at Cardiff University. The observers with protanopia and deuteranopia had their colour vision deficiency confirmed using the Neitz anomaloscope. Other observers had their ocular health status confirmed after pupil dilation by a clinician who examined the retina using binocular indirect ophthalmoscopy, fundus photography and optical coherence tomography (OCT). The characteristics of the patients are described in Table 1 in the Supplemental Digital Content.

**Figure 1:**
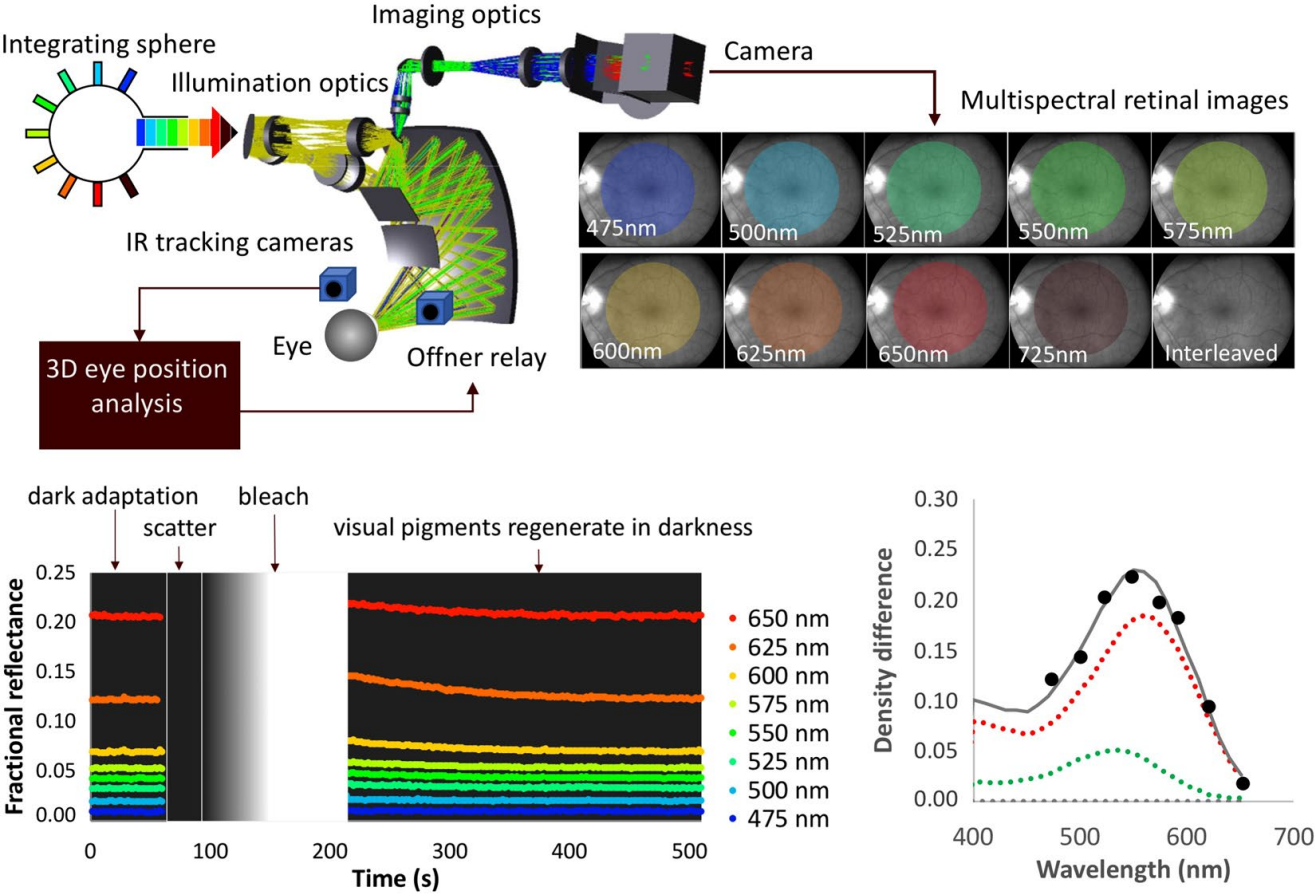
Densitometer schematic and initial workflow. (Top left) Light from one of 9 LEDs, which are driven sequentially at 5 Hz, is projected into an integrating sphere to ensure uniformity before passing into the illumination optics. Offner relay optics controlled by an infrared (IR) eye tracking system ensures continuous optical alignment with the participant’s eye. (Top right) Multispectral retinal images are captured in low light levels. (Bottom left) A timeline showing the sequence of events (dark adaptation period, scatter measurement, bleach and visual pigment regeneration) together with typical reflectance data obtained during the corresponding periods superimposed. (Bottom right) Example density difference data (black symbols) obtained from a single pixel at a single point in time fitted with a spectral profile (solid grey curve) which describes the sum of rod, m-cone and l-cone density difference spectra (grey, green and red dotted curves respectively). In this example, the data were collected from the fovea 6 s after the bleach and calculated using equation 2 (see Supplementary Digital Content).

The design of our multispectral imaging densitometer and initial workflow are shown in Fig. 1. A set of mirrors in the form of an Offner relay create an image of the pupil of the eye. An annular mirror located at this pupil plane is used to project the illumination light into the eye through an annular pupil while allowing light from the centre of the pupil to be transmitted to the densitometer camera to form a retinal image. Eye position is measured in three dimensions on the basis of images from two IR cameras running at a frame rate of 40 Hz. The centre of the pupil is located using a real time random sample consensus (RANSAC) approach to locate the pupil centre, and trigonometry. These measurements drive the Offner relay optics to maintain continuous optical alignment of the instrument pupil to the pupil of the eye.

A bank of 9 light emitting diodes (LEDs) housed in an integrating sphere and operated sequentially at 5 Hz, provided the multispectral illumination. Retinal illuminance was minimised (2.9 log scot trolands) to avoid significant photopigment bleaching by exploiting the sensitivity of an Andor Luca-R camera. Over a 10 minute recording the measuring sources bleached approximately 1.5% of cone and 3.0% of rod visual pigment^20,21^. The participant’s fixation was aided with a blue (450 nm) fixation cross (subtending 5° diameter at the eye) located at the centre of the imaging field. A pair of edge pass filters were fitted in front of the camera to block light from the fixation target and from the infrared LEDs used for the pupil tracking system. Light from an additional ‘white light’ LED passed through a 3 mm thick GG455 glass filter, to attenuate blue light, was used to bleach visual pigment. This source was set to deliver a retinal illuminance of 6.1 log scot td which bleached approximately 95% cone and 98% rod visual pigment over the 60 s bleaching period^20,21^. This is a lower estimate for bleaching because this retinal illuminance was only reached after a 60 s ramp so as to improve participant comfort.

During most operations the illumination and imaging fields were coincident and subtended ~20° at the eye. However, during backscatter measurements an additional vertical bar mask was introduced into the illumination field. The raw images produced by the system were corrected for charge-coupled device (CCD) bias, stray light and back scatter. Calibrations against a ‘white’ PTFE surface were used to derive absolute reflectance measurements from the retina in dark adapted and post bleach conditions.

All time dependent functions were synchronised by a transistor–transistor logic (TTL) pulse from the Andor Luca-R camera, and operation of the entire system was coordinated by a tailor-made mother board controlled using the LabView software environment. A full description of the device, image processing steps, fitting procedures, system calibrations and benchmarking is provided in the Supplemental Digital Content.

After recording any relevant ocular history, and an eye examination, one drop of 1% Tropicamide was instilled into each eye to dilate the participant’s pupils. While the drops took effect and the eye recovered to its dark adapted (DA) state, participants waited in a dim (<1.5 log scot td) room for 30 minutes. Participants then sat in front of the IRD with their head placed on a conventional ophthalmic head rest, and the eye tracking system was engaged. The timeline and individual steps of a functional imaging sequence are shown in Fig. 1 (bottom left). Typically, this involved the recording of: 1) a dark adapted (DA) image sequence for 1 min, 2) an assessment of back scatter (30 s), 3) a high resolution fundus photograph, 4) a long duration photopigment bleach (120 s) that included a 60 s minute ramp to ensure participant comfort and, 5) a bleach recovery (BR) recording that lasted 5–20 min depending on the exact protocol.

The results generated by IRD were in the form of topographical heat maps describing the distribution of rod and cone pigments and their synthesis rates across the central retina (21° diameter). A fundus photograph taken during the recording procedure was used to cross reference pigment metrics with the underlying retinal pathology. Descriptive statistics were used to summarise optical density and recovery rate metrics for those with and without AMD.

## Results

**Figure 2:**
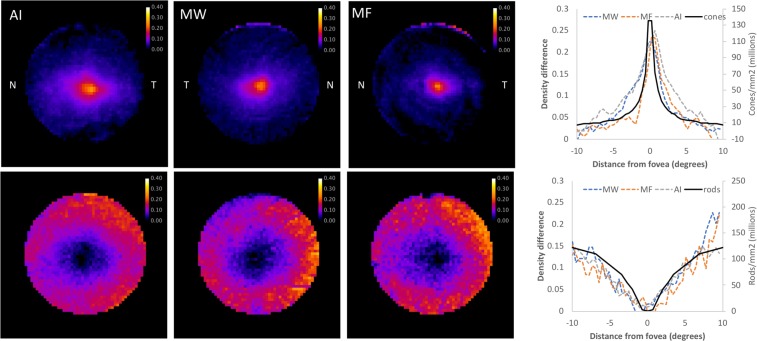
Topographical variations in cone and rod photoreceptor density. (Top left) Heat maps describing the spatial distribution of cone visual pigment in three healthy observers with the nasal and temporal retina indicated by the letters N and T respectively. (Top right) Plot showing the optical density of cone visual pigment across a horizontal section of the retina for the same three observers. (Bottom left) Heat maps describing the spatial distribution of rod visual pigment in the same observers. The circular heat map images are 21°of visual angle in diameter. (Bottom right) Rod photopigment distribution along the horizontal meridian increases toward the edge of the image. In the plots, the solid black curves describe photoreceptors density determined on the basis of histology^22^.

The optical density maps produced by IRD correspond to the known distributions of rod and cone photoreceptors. Figure 2 describes the topographical distribution of rod and cone visual pigments across the central retina in 3 healthy older observers (69, 81 and 82 years of age), and the accompanying horizontal density profiles show that the measured visual pigment density largely mirrors histological estimates of rod and cone photoreceptor packing density^22^.

Next we considered the temporal aspects of photopigment regeneration. Although the regeneration of visual pigment has been modelled using first-order expressions for many decades, more recently it has been suggested that rate-limited kinetics provide a superior description^1,23^.

**Figure 3:**
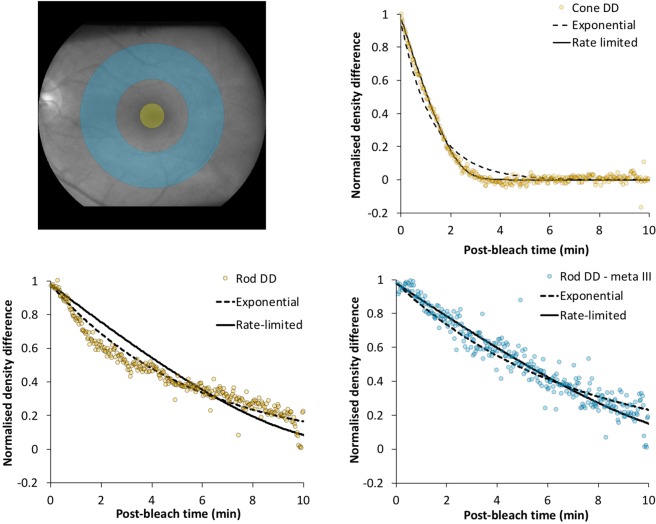
Analysis of the temporal characteristics of visual pigment regeneration for subject O.S. (Top left) Fundus photograph showing areas where cone (yellow circle) and rod (blue annulus) data were extracted. (Top right) Density difference as a function of time after the bleach for cone data. The rate limited function (see equation 10 in Supplementary Digital Content) provides a visibly better fit than the exponential function (equation 9). (Bottom left) Post bleach density difference data for rods (orange symbols) shows an inflection about 2 min after the bleach. The solid and dashed curved lines described the best fitting rate limited (equation 10) and first order (equation 9) functions respectively. Neither expression is a particularly good fit to the data. (Bottom right) By allowing the spectral fitting routines to extract a component with the spectral signature of metarhodopsin III (equation 7) the inflection in the data is removed and the rate-limited function now provides a superior fit to the data. With *K*_*m*_ and B fixed at 0.2 and 0.98 respectively, *v* = 0.097 min^−1^.

Cone data extracted from the central 4° clearly show that rate-limited behaviour provides a superior description of the recovery (see the smooth curve through the yellow data points in the top right panel of Fig. 3). With the value of *K*_*m*_ in equation 10 (see Supplementary Digital Content) fixed at 0.2 and *B* at 0.95, the maximum recovery rate was *v* = 0.47 min^−1^. However, examination of rod density difference data from a more peripheral annulus (7°–10° inner-outer radius) suggests the recovery profile obtained via IRD is more complicated (see orange circles in Fig. 3, lower left hand panel). Superficially the exponential function (dashed line) appears to provide a superior description; however, careful examination shows a clear change in recovery rate approximately 2 min post bleach. We attribute this inflection to metarhodopsin III. By exploiting the power of multispectral imaging we were able to tease apart the absorption spectra of the post bleach photoproduct metarhodopsin III from rod and cone visual pigment spectra.

**Figure 4:**
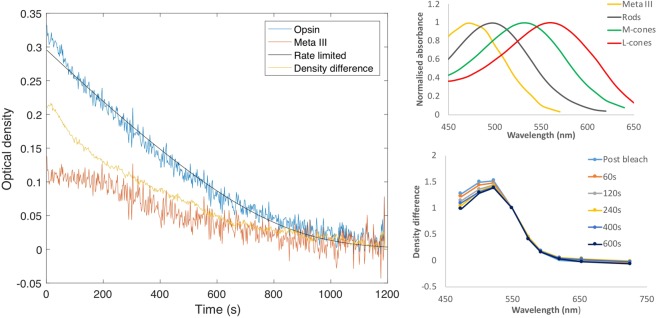
Analysis of spectral data from the temporal retina (8.5°) of subject T.M. who has negligible cones at this location. (Left) Spectral density difference data (here summarised by the peak difference) obtained at each point in time after the bleach (yellow) were fitted with rhodopsin and metarhodopsin III pigment spectra according to equation 8. This resulted in estimates for the optical density of metarhodopsin III (orange) and the rhodopsin density difference (blue). The fitting routines were implemented off line using the fminsearch function in Matlab. The solid black curve is equation 11 with *K*_m_ = 0.20, *B* = 0.98 and *v* = 0.079 min^−1^. (Top right) Visual pigment spectra used in the fitting routines. (Bottom right) Density difference spectra obtained at different times in the dark normalised at 550 nm. Note the subtle change in shape observed for the shorter wavelengths which we attribute to differences in the decay rate of opsin and metarhodopsin III.

For example, the data in Fig. 4 were obtained from the temporal retina (~10°) of an observer who had no measurable cone optical density (OD) at this location. It can be seen that the post bleach optical density of metarhodopsin III is significant, approximately 1/3 that of opsin, and that the latter part of the decay profile is similar to that of opsin. These observations corroborate earlier findings by Ripps and Weale (1969, their Fig. 4)^24^. Relative to the dark adapted state, the density difference measured at any point in time after the bleach (yellow line in Fig. 4, left panel) is dependent on the optical density of rhodopsin (here described by its bleached fraction opsin) and metarhodopsin III. The relative stability in the concentration of metarhodopsin III immediately post bleach (red line in Fig. 4, left panel) means that the recovery observed shortly after the bleach is due almost entirely to the decay of opsin. Thereafter, the density difference appears to decay more slowly because the increasing optical density attributed to the accumulation of rhodopsin is, to some extent, offset by the loss of metarhodopsin III.

Returning to the rod data presented in Fig. 3 (Bottom left), removal of metarhodopsin III’s contribution to the post bleach density difference data largely removes the inflection (see blue dots in Fig. 3 bottom right; analogous to the plot of opsin decay shown in Fig. 4). Once corrected for metarhodopsin III, the rate-limited function (equation 10) provides a superior fit to data; with *K*_*m*_ and B fixed at 0.2 and 0.98 respectively, the recovery rate is estimated to be *v* = 0.097 min^−1^.

**Figure 5:**
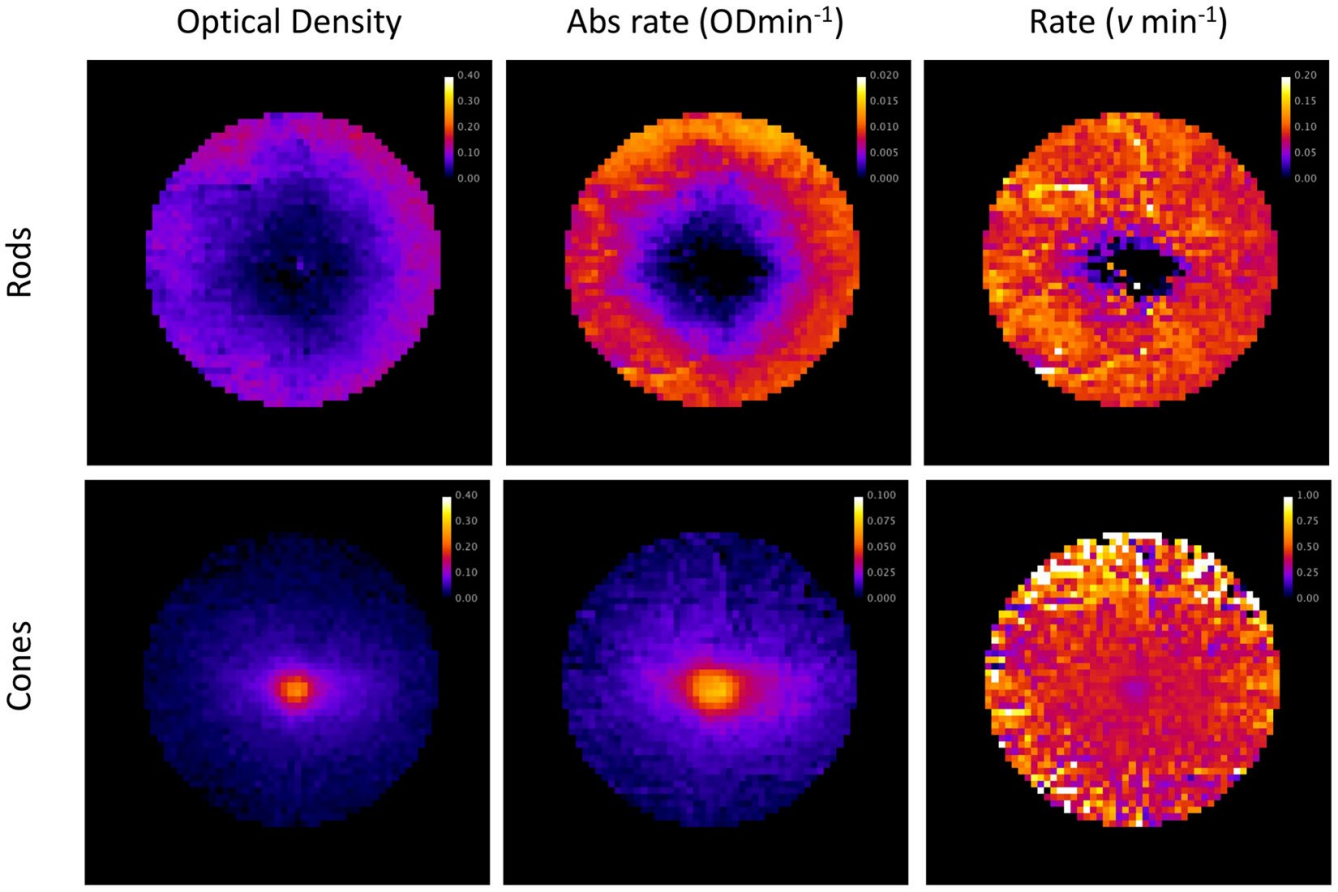
Heat maps for subject J.F. describing the spatial distribution of rod (upper row) and cone (lower row) visual pigment characteristics. (Top left) Rod density difference described in optical density units. (Top centre) Absolute rod recovery rate described in ODmin^−1^. (Top right) Rod recovery rate per molecule expressed in terms of *v* min^−1^. Outside the central 6° degrees the recovery rate is relatively uniform; with *K*_m_ = 0.2 and *B* = 0.98, *v* = 0.095 SD 0.021 min^−1^. (Bottom left) Cone density difference peaks in the central retina. (Bottom centre) The peak recovery rate for cones at the very centre of the retina (0.073 OD min^−1^) is approximately 8 times faster than that of rods. (Bottom right) In comparison to the absolute recovery rate, the cone recovery rate per molecule (*v* = 0.403 SD 0.035 min^−1^ with *K*_m_ = 0.2 and *B* = 0.95) is relatively uniform across the central 6°.

Topographically, absolute cone and rod recovery rates vary significantly across the retinal surface, and this variation maps onto the spatial distribution of the pigments. For example, the heat-map showing the spatial distribution of rod pigment in a typical control participant in Fig. 5 (Top left) has a similar appearance to that of the absolute recovery rate shown in Fig. 5 (Top centre). There is a clear donut appearance, with the absolute regeneration rate being greater at the edge of the image (typically 0.009 OD min^−1^) where rod density is high. In the centre of the image, where rod density drops precipitously, the observed recovery rate drops to noise levels. To avoid conflating amplitude with time-course, the absolute recovery rate is divided by total density difference to provide a measure of the recovery rate per molecule in terms of *v* (see Fig. 5 top right). In comparison with the absolute regeneration rate, the recovery rate per molecule is relatively consistent, except for the very centre of the image where values for *v* tend toward noise levels because of the vanishingly low optical density of rhodopsin in this part of the retina; outside the central 6° (diameter), *v* = 0.095 SD 0.021 min^−1^.

For cones, optical density and absolute recovery rates peak in the central retina with the absolute peak rate (0.073 OD min^−1^) being about 8 times greater than for rods (Fig. 5 bottom row). In comparison, the recovery rate per molecule is relatively uniform across the central 6° (*v* = 0.403 SD 0.035 min^−1^).

Given that the outer retinal complex is involved in the pathogenesis of AMD and the RPE is also responsible for the production of 11-cis retinal, the rate-limiting step in rhodopsin synthesis, rod and possibly cone visual pigment synthesis rates are slowed in people with this eye condition. To explore the potential of IRD as a marker of outer retinal complex function in AMD we recruited 5 people with intermediate AMD (average age 72.8 SD 7.0 years) and 5 age matched controls (average age 76.0 SD 6.3 years). The characteristics of participants with AMD are described in Table 1 (see Supplementary Digital Content).

**Figure 6:**
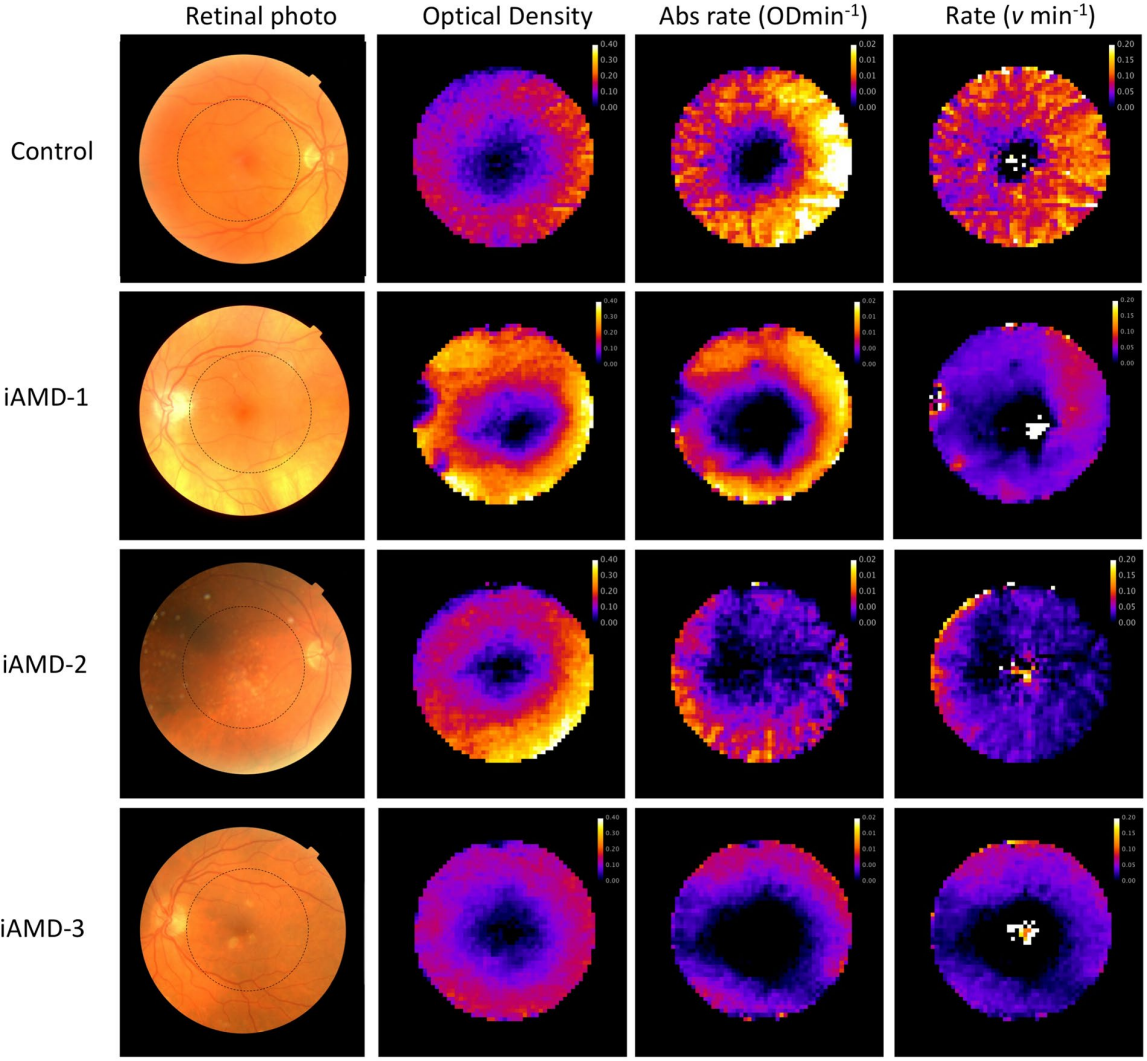
Functional imaging of the outer retinal complex in a typical healthy age matched control and in three people with intermediate age-related macular degeneration (iAMD). Images in the first column show traditional colour fundus photographs and the area of interest studied by IRD (highlighted by a dashed circle). Images in the second column are optical density maps for the rod visual pigment rhodopsin. Heat maps in the 3^rd^ column describe the absolute recovery rate of rhodopsin over the same area. In the 4^th^ column the heat maps describe the recovery rate per molecule (the parameter *v* in equation 10). Data toward the centre of these images are noisy because the total optical density and regeneration of rhodopsin in the central retina are negligible.

Topographical maps of rod optical density, absolute photopigment recovery rates (OD min^−1^) and rates per molecule (*v* min^−1^) are shown for 1 healthy control and 3 people representative of those with AMD in Fig. 6. On average, the total optical density of rod and cone visual pigments was consistent with that found in healthy controls, but recovery rates were reduced.

The pattern of loss was not consistent between people with AMD, and did not always map onto features observed in the fundus photographs. For example, the person identified as iAMD-1 shows elevated rod optical density levels throughout the peripheral retina, but the recovery rate per molecule is clearly reduced, except for a small patch of retina toward the upper right-hand side of the image. The person identified as iAMD-2 again shows a relatively normal rod optical density, but the recovery maps have a mottled appearance. In the person with large soft drusen toward the fovea (iAMD-3), rod optical density is somewhat reduced across the retina and there is no measurable recovery toward the centre of the retina (the speckled appearance in the very centre of the image is noise). With advancing pathology (going down Fig. 6) both the absolute recovery rate and the rate per molecule tend to decline, with an enlarged central area showing no measurable recovery.

**Figure 7:**
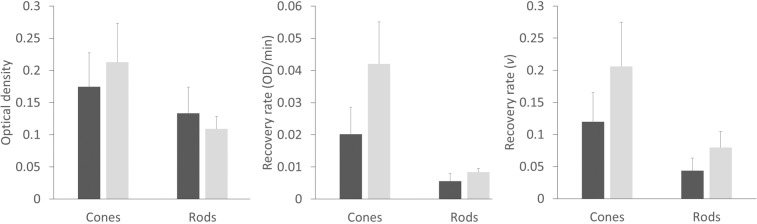
Average data obtained from 5 people with (dark grey) and 5 people without (light grey) AMD. (Left) Optical density of cone visual pigments measured at the fovea and rods in a peripheral annulus for those with and without AMD. (Centre) Absolute recovery rates for rod and cone visual pigments at the same locations showing a marked reduction in absolute cone recovery rate in those with AMD. (Right) Slowed rod and cone recovery rates per molecule (*v*) in people with AMD. Error bars describe the standard deviation.

The average data obtained from those with and without AMD are summarised in Fig. 7. The cone data is extracted from the fovea (0.9° diameter) and the rod data from within an annulus (6°–8° inner-outer radius). For rod photoreceptors, the recovery rate per molecule in healthy older adults was *v* = 0.079 SD 0.024 min^−1^ and for cones *v* = 0.206 SD 0.069 min^−1^. For those with AMD the recovery rates per molecule were slower, with average values for rods and cones being *v* = 0.043 SD 0.019 min^−1^ and *v* = 0.119 SD 0.046 min^−1^, respectively.

## Discussion

High fidelity IRD is a powerful new technique that will help researchers and clinicians probe outer retinal physiology. Significantly, the technique delivers two useful pieces of information. Firstly, it maps rod and cone optical density, and in so doing acts as a marker of photoreceptor outer segment shortening, degeneration or loss. Secondly, by quantifying visual pigment synthesis rates it provides topographical information about the functional integrity of the outer retinal complex. Combined, these two pieces of information can help identify areas within the macular region where photoreceptors are present but where outer retinal complex function is compromised. This information is significant because the outer retinal complex and in particular the RPE is implicated in many blinding eye conditions, including age-related macular degeneration, and there are no other ways of probing RPE function on a point by point basis across the retinal surface.

Following initial benchmarking (see Supplementary Digital Content) we set out to map rod and cone optical density in healthy controls. Although the topographical distribution of visual pigment density largely mirrors histological estimates of rod and cone photoreceptor packing density^22^, there is a subtle mismatch at the fovea (see Fig. 2). The cone OD profile has a broader shape at the fovea, which is likely to reflect the fact that the cones there are closely packed and the outer segment length increases in the central fovea^25^. Despite this subtle difference, visual pigment optical density will be a useful marker for outer segment shortening, degeneration or loss, even if it does not provide a direct measure of photoreceptor packing density.

By maintaining close alignment with the eye over extended periods of time, IRD has allowed us to probe the time course of visual pigment regeneration with considerable precision. Post bleach density difference data strongly support the suggestion that cone visual pigment regenerates according to rate-limited behaviour, and, once corrected for metarhodopsin III, rhodopsin regeneration behaviour is also consistent with rate-limited regeneration (see Fig. 3). Our data suggest that the presence of metarhodopsin III distorts the raw density difference data, and this may have similarly affected previous densitometric measurements of rhodopsin regeneration^15,19,26^. Even at 530 nm the optical density of metarhodopsin III is still significant, being ~30% of its peak value^27^. On the other hand, Alpern (1971) substantially avoided the effect of metarhodopsin III by making measurements at 555 nm where the optical density of metarhodopsin III drops to less than 10% of its peak value^6^. Had we failed to correct for the distorting effects of metarhodopsin III, and fitted the rate-limited model to the raw density difference data, our estimate for the rate would have been ~10% faster (*v* = 0.109 min^−1^). Although spatial averaging allows us to trace the products of rhodopsin photolysis *in vivo*, this is not possible on a pixel by pixel basis because of the reduced signal to noise ratio, and hence our point wise estimates of recovery rates are based on the raw rod density difference data and include the modest error described, likely overestimating the true rhodopsin recovery rate by 10%.

The heat maps describing rod and cone pigment synthesis rates in Fig. 5 exemplify the functional imaging capability of IRD. For the rods, the topographical visual pigment synthesis maps provide a measure of RPE function because the regeneration of visual pigment in these photoreceptors is determined by the RPE’s ability to supply 11-*cis* retinal. The fact that cones can access two independent visual cycles, one based in the RPE and one in the Müller cells, complicates the interpretation of cone regeneration data^2^. Both RPE and Müller cell pathology may impact on cone pigment synthesis rates. Evidence from amphibians suggests that cone pigment regeneration rates attributable to the RPE and Müller cell pathways are roughly equal^28^; however, there are currently no data on the relative importance of these systems in humans. Indeed, it seems possible that the relative importance of these two systems to cone visual pigment synthesis rates might vary with eccentricity. The RPE ‘visual cycle’ being relatively more important at the very centre of the retina, where cones have exclusive access to the RPE and where typical Müller cells exist alongside specialised Müller cells with both glial cell types extending from the internal limiting membrane to the outer limiting membrane^29^. Our observation of slowed cone pigment synthesis rates at the fovea of people with AMD supports the notion that the RPE visual cycle has a major role to play in the regeneration of visual pigment at the human fovea (see Fig. 7).

The results from the small number of people with AMD examined here highlight the clinical potential of the technique. By mapping visual pigment synthesis rates, previously unknown regional differences in outer retinal complex function are revealed, even in early stage disease (Fig. 6). The functional imaging delivered by IRD supports the use of ‘dark adaptation’ metrics in the assessment of AMD, but also highlights some of the limitations associated with current psychophysical approaches. Dark adaptation is a leading biomarker for AMD^30,31^, but data are typically only obtained from a single retinal location, measurement time is relatively protracted, and results between individuals with a similar disease status can be variable^31^. The differences in topography of visual pigment synthesis rates between individuals with AMD revealed by IRD highlight the problem of collecting data from a single region of the retina, and may also provide an explanation for the significant between-subject differences in dark adaptation metrics that have been observed^31^.

The technique of retinal densitometry, first described in the 1950s, has been plagued by persistent technological hurdles that have, until now, prevented the technique from reaching its full potential^7^. Previous investigators have mapped total visual pigment optical density *in vivo* using video systems or scanning laser ophthalmoscopes^16,17,19^ and some have used imaging devices to trace the regeneration of rhodopsin from discrete retinal locations^15,32^. However, to our knowledge this is the first time that both visual pigment distribution and regeneration rates have been mapped across the central retina. In so doing, the approach to imaging retinal densitometry described here represents a step change in performance. For the first time, retinal densitometry delivers functional imaging of the outer retinal complex.

Precise and continuous optical alignment between the eye and densitometer optics is a vital prerequisite for outer retinal functional imaging. Preliminary investigations with a more conventional densitometer design based around the optics of a fundus camera and bite bar (not shown here) helped us understand the exquisite sensitivity of retinal reflectance measurements to pupil alignment errors as small as ~0.1 mm. Even in well trained subjects, maintaining optical alignment with a conventional bite bar proved challenging, with recordings often showing small random fluctuations in retinal reflectance. Previous densitometry techniques have endeavoured to correct for these artefactual temporal fluctuations by scaling reflectance measurements in the visible range (400–650 nm) according to a near infra-red (NIR) reference measurement^6^. However, there are two reasons why this approach is flawed. Firstly, the eye suffers from significant chromatic aberration, and therefore fluctuations in long wavelength reflectance rarely mirror fluctuations at shorter wavelengths. Secondly, recent evidence suggests there may be physiological fluctuations in NIR reflectance^10,14^, possibly arising from changes to optical path length^33^, that confound its use as a reference. Our solution was to employ an optical approach more usually found in the field of astronomy to ensure continuous, precise optical alignment throughout the recordings. That is, by driving Offner relay optics according to a feed-forward IR pupil tracking system, we were able to maintain precise optical alignment indefinitely and very nearly eliminate temporal instability in reflectance measurements (see Supplementary Digital Content).

Another significant innovation here is the introduction of a scatter correction that improves the fidelity of visual pigment OD estimates. Previously, light scattered back from the eye’s optical media has resulted in a wavelength dependent attenuation of the measured signal^8^. In conventional optical systems, particularly those with a large field of view, this has manifested itself in a spectral shift toward longer wavelengths^17^ which has thwarted attempts to obtain precise estimates of visual pigment OD. Several elegant retinal reflection models have been developed that can be used to characterise the optical properties of retinal components including the optical density of visual pigments^18,34,35^. Our preliminary work (not shown here) included spectral modelling along the lines suggested previously. Whilst this approach produces plausible estimates which described the optical characteristics of the eye’s main pigments (blood, melanin, macular pigment, and optical media) the number of free parameters in the models made fitting problematic. Our solution was to implement a two stage scatter correction, the first correcting for back scatter from the ocular media and the second for back scatter from the retinal nerve fibre layer (see Supplementary Digital Content). The utility of this relatively simple approach was confirmed by data from 2 observers who possess only a single type of visual pigment at the fovea (see Supplementary Digital Content).

Although the IRD technique described here provides a step change in the assessment of outer retinal function there are some limitations. The current technique does not attempt to measure the OD for short or blue light wavelength sensitive cones (S-cones). S-cones are absent from the fovea but constitute about ~7% of the total cone population at greater eccentricities, and will undoubtedly have an impact on retinal reflectance at shorter wavelengths^36^. The spatial resolution of the system, currently 130μm, is limited by the sensitivity of the Andor Luca-R camera, by the optical design of the illumination and imaging pupils, and by our desire to keep photopigment bleaching to negligible levels (no more than 3%). Although functional imaging at this level of resolution is useful clinically, the images produced are coarse relative to conventional fundus photography. The decision to fix the amplitude of the second scatter correction, on the basis that normal variations in the thickness of the retinal nerve fibre layer (RNFL) are modest, introduces another potential source of error. We estimate the error in our optical density estimates attributable to this assumption to be no more than 5% in normal participants^37^. Another parameter that could be improved still further is the recording time, which is typically 5–10 minutes. Theoretically, the precision of photopigment recovery rate estimates should be limited by photon shot noise only, but in reality, and despite the high fidelity eye tracking, small temporal transients, due to fixation losses and blinks, dictate the recording duration to ensure precise rate estimates.

In conclusion, the technique of IRD is able to map rod and cone visual pigment synthesis rates and their absolute optical densities across the central retina. The use of 3D eye tracking to drive relay mirror optics facilitates precise retinal reflectance measurements over time that would otherwise be contaminated by minute eye movements. This, combined with a correction that compensates for wavelength dependent scatter in the ocular media and multispectral imaging, ensures that the total optical density of the visual pigments and their absorption spectrum is recovered with high fidelity. We anticipate that this new technique will help researchers and clinicians study outer retinal physiology in both health and disease. Results from the people with AMD studied here are distinct from those of age matched controls, and hence IRD will likely be a useful functional biomarker for monitoring disease onset, progression and treatment interventions.

## Supplementary information

Supplemental Digital Content.

## Data Availability

The datasets generated during and/or analysed during the current study are available from the corresponding author on reasonable request.
