# Supplementary material for: Functional Imaging of the Outer Retinal Complex using High Fidelity Imaging Retinal Densitometry
Source: Sci Rep. 2020 Mar 11;10:4494. doi: 10.1038/s41598-020-60660-9 (PMC7066170; doi:10.1038/s41598-020-60660-9)
Supplement: Supplementary file 1 — Supplemental Digital Content. [file 41598_2020_60660_MOESM1_ESM.pdf]

# Functional Imaging of the Outer Retinal Complex using High Fidelity Imaging Retinal Densitometry

Tom H Margrain[1], David Atkinson[2], Alison M Binns[3], James Fergusson[1], Allannah Gaffney[1], David Henry[2], Chris Jones[1], Trevor D Lamb[4], Dave Melotte[2], Chris Miller[2], Stephen Todd[2], Ashley Wood[1]

**Affiliations**:
[1]School of Optometry and Vision Sciences, Maindy Road, Cardiff University, Cardiff, Wales, UK CF24 4HQ

[2]UK Astronomy Technology Centre, Royal Observatory, Edinburgh, Blackford Hill, Edinburgh, UK, EH9 3HJ

[3]School of Health Sciences, City, University of London, Northampton Square, London, EC1V 0HB

[4]Eccles Institute of Neuroscience, John Curtin School of Medical Research, The Australian National University, Canberra, ACT 2601, Australia

**Benchmarking investigations:** To benchmark the performance of the system we first applied our IRD technique to recover the wavelength dependent optical density (OD) of a known, visual pigment like filter (Kodak Wratten CC10M) mounted in a model eye (see Figure S1). The model eye, based on the optical design proposed by Gullstrand was 3D printed using the liquid photopolymer R11 (EnvisionTec, Inc.), the 'cornea' had a refractive index of 1.4 and had a front surface curvature of 7.8mm, a rear surface curvature of 6.7mm and centre thickness of 0.5mm. The crystalline lens also had a refractive index of 1.4 and had a front and rear curvature of 10.0mm and -8.0mm respectively. The inner surfaces of the eye were painted with a matt acrylic paint (Vallejo flat red 70818) whose reflectance profile mimicked that of the human eye. When filled with water, the eye had a focal length of 15.9mm. The optical density of the filter was determined using IRD by obtaining retinal reflectance measurements from the model eye with and without the filter positioned anterior to the retinal surface. These measurements were adjusted for back scatter from the eye's optical media by subtracting the signal contained in a non-illuminated vertical bar that was projected onto the 'retinal' surface (see scatter correction below). The OD was calculated as the logarithm of the (without filter – scatter signal) / (with filter – scatter signal) reflectance ratio. The recovered spectrum was a good fit to that determined by a calibrated spectrometer ($R^2$=0.993) (see Bottom right of Figure S1).

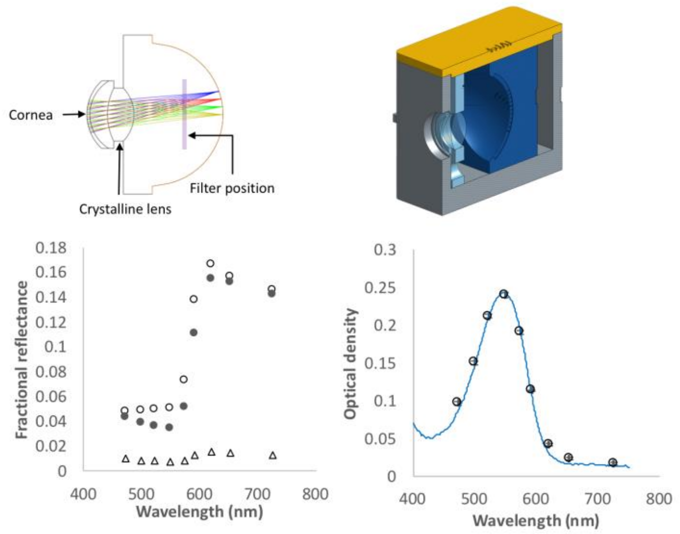


***Figure S1.*** *Model eye construction and recovery of the absorption spectra of a known visual pigment like filter. (Top left) The optical design of the model eye used to benchmark the system was based on that proposed by Gullstrand. The location of the visual pigment like filter (Kodak Wratten CC10M) is highlighted. (Top right) Computer aided design (CAD) drawing of model eye in cross section. (Bottom left) Reflectance signal obtained from the illuminated retina with and without the visual pigment like filter (filled and unfilled circles respectively). The 'scatter signal' contained at the centre of a non-illuminated bar mask (triangles) which was subtracted from the with / without filter signals before calculating optical density. (Bottom right) Optical density of the visual pigment like filter determined by an Ocean Optics Flame spectrometer (blue curve) and that recovered by IRD (open symbols). Error bars (approximately the size of the symbols) describe the SE.*

**Eye tracking and scatter correction:** We next evaluated the pupil tracking system and scatter correction method in human observers *in vivo*. Inspection of the time dependent reflectance data with and without the eye tracking system engaged, revealed the extent to which the Offner relay system removes eye movement related temporal noise (see Figure S2). When the tracking system was turned off, substantial variations in retinal reflectance were observed (coefficient of variation (CoV) ~220%). This temporal instability was reduced about 30 fold when the tracking system was engaged (CoV ~8%), with the residual variability being mainly attributable to photon shot noise. The Offner relay eye tracking system typically reduced optical alignment errors from ~2.4mm (SD) to ~0.17mm (SD).

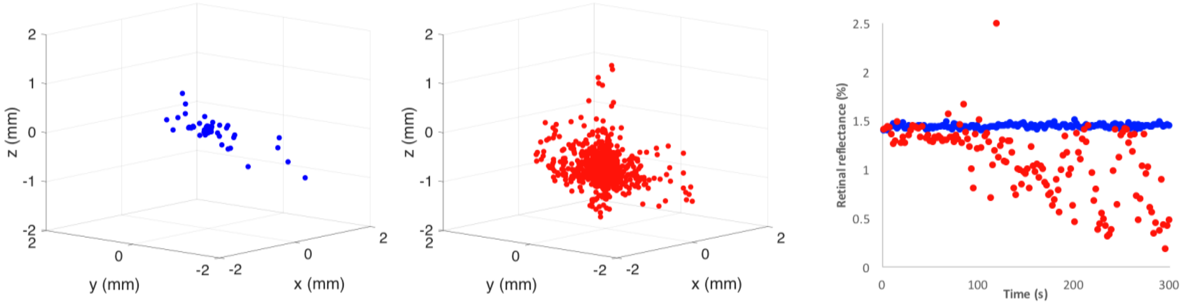


***Figure S2.*** *Effect of eye tracking system on optical alignment and retinal reflectance measurements. (Left) Position error between the centre of a human observer's pupil and the optics of the imaging system with the IR Offner relay tracking system turned ON (note there are 3000 mostly superimposed, data points in this plot). (Centre) Position error with the tracking system turned OFF. (Right) Central retinal reflectance at 550nm with tracking ON (blue) and OFF (red).*

Having eliminated much of the temporal noise, we set out to benchmark the ability of IRD to recover visual pigment spectra *in vivo*. To simplify the analysis the observers who took part in this investigation had known colour vision deficiencies. That is, one was a protanope who possessed only 'M-cones' at the fovea; the other was a deuteranope whose fovea comprised only 'L-cones'. In the human eye, a two stage scatter correction was implemented to remove the effects of back scatter. The first stage was identical to that described for the model eye i.e. the subtraction of the signal returning from a non-illuminated vertical strip (see Figure S3). The second scatter correction, derived from optical coherence tomography images, corrected for topographical differences in the thickness of the retinal nerve fibre layer (see Figure S3). In biological tissues scatter typically declines monotonically with increasing wavelength according to a Mie scattering function[1]. However, examination of the signal at the centre of the non-illuminated strip, where we might expect back scatter to dominate, shows a relatively complex picture (see Figure S3 top right). The signal recorded drops from about 475nm to 550nm but increases significantly for wavelengths >575nm. We attribute this spectral profile to two distinct sources. At shorter wavelengths the signal observed in the non-illuminated strip is dominated by scatter originating from the optical media, which we assume to have a Mie scattering profile. This is because the optical density of retinal pigments such as melanin and blood are relatively high at these wavelengths, which minimises direct retinal reflections but scattering from the media is at its peak. At longer wavelengths, beyond 575nm, the increase in signal is mainly due to the precipitous reduction in the OD of blood which becomes nearly transparent at wavelengths >600nm. By only subtracting the optical media related Mie scatter component from the reflectance data we apply a correction that shifts the spectra to shorter wavelengths. A second scatter correction that also had a Mie scattering spectral profile, but which varied topographically, according to the spatial distribution of the retinal nerve fibre layer (RNFL) was also applied. This second correction brought the spectral profile obtained from the nasal retina, where the RNFL is significant, into register with that obtained from the temporal retina where scatter originating from the RNFL was assumed to be negligible.

We reasoned that if the scatter correction algorithm worked the visual pigment fitting routine would attribute the measured density difference to M-cone and L-cone pigments only at the fovea in the protanope and deuteranope respectively. In the temporal retina of the same individuals, approximately 7°-10° (inner-outer radius) from the fovea, we anticipated that the density difference spectra recovered 3 minutes after bleach cessation would be dominated by the rod visual pigment rhodopsin, albeit not at peak density, because rods outnumber cones by about 10 to 1 at this retinal eccentricity and cone pigments have largely regenerated by this time[2]. Hence, the cone contribution to the density difference spectra measured at this location and point in time should be vanishingly small. We also anticipated that the bleaching photoproduct metarhodopsin III, whose absorbance peaks around 475nm, may shift the measured spectra to longer wavelengths than might be expected on the basis of data obtained via microspectrophotometry (msp)[3-6].

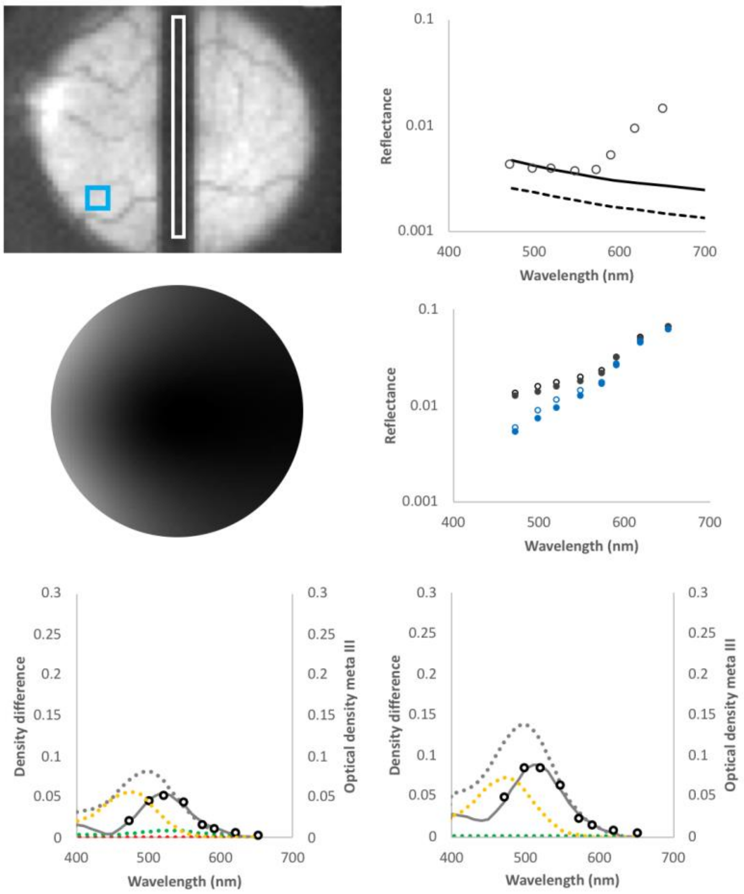


***Figure S3.*** *Steps involved in the adjustment of visual pigment spectra for two types of scatter. The first originated from the eye's optical media the second from the retinal nerve fibre layer (RNFL). (Top left) Scatter from the eye's optical media was determined by projecting a vertical bar mask onto the retina and measuring the signal contained in the non-illuminated vertical strip (within the elongated white rectangle). The signal contained in the bar mask region was spatially uniform but varied spectrally. (Top left) Spectral distribution of light within the bar mask (black circles), obtained before the bleach, and the Mie scatter function which characterised light scattered back from the optical media (solid black curve). The dashed black curve describes a second Mie scattering function designed to correct for light reflected back from the RNFL. This varied both spectrally and spatially. (Middle left) Grey scale image describing the spatial distribution of light scattered back from the retinal nerve fibre layer. The pattern was based on optical coherence tomography (OCT) images of the RNFL and its amplitude was fixed so that the spectra recovered from the nasal retina were the same as those from the temporal retina where we assumed scatter from the RNFL to be negligible. (Middle right) Raw (black symbols) and back scatter corrected (blue symbols) retinal reflectance data obtained from that part of the retina highlighted by the blue box shown in the fundus photograph (Top left). Filled symbols describe data from the dark adapted retina and open symbols data from the bleached retina. (Bottom left) Analysis of the uncorrected density difference data (open symbols) obtained 3 minutes after the bleach derived using equation 2. At this point in time, the density difference (the ratio between the measured retinal reflectance and the average measured during the dark adaptation period) attributable to cones should be zero because cone visual pigment levels have recovered to their dark adapted level. However, without the scatter correction the pigment fitting routines have, erroneously, identified un-regenerated M-cone pigment (green dotted curve) as well as un-regenerated rhodopsin (grey dotted curve) and provide a relatively low estimate of the total density difference of 0.05 (solid grey curve). (Bottom right) Application of the two stage scatter correction shifts the spectra to shorter wavelengths, eliminates the erroneous M-cone signal, and boosts the density difference to 0.09. The orange dotted lines (see Bottom row) describe the post bleach optical density of meta III which was required to bring the fitted density difference spectra into register with the data.*

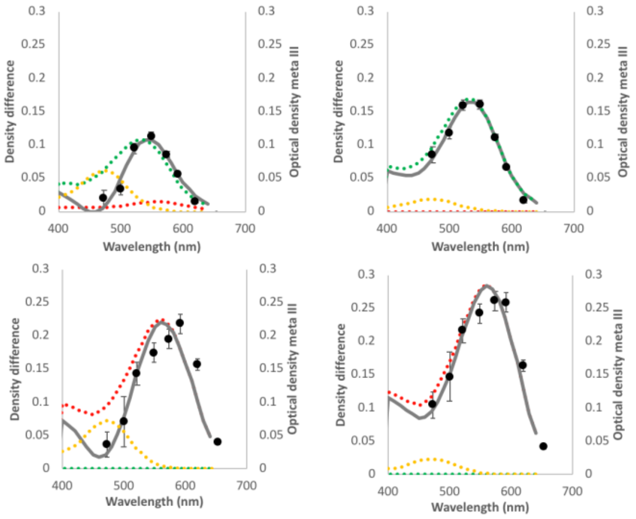


***Figure S4.*** *Density difference data obtained at the fovea immediately after the bleach from an observer with only M-cones (top row) and from an observer with only L-cones (bottom row). The data in the left hand column describes the raw density difference data and the data in the right hand column has been corrected for back scatter (filled symbols). The solid grey curves are the least squares fits based on M-cone and L-cone pigment density differences and the optical density of meta-iodopsin III, shown as green, red and orange dotted curves respectively (see equation 6). (Top left) Without correcting for scatter, the best fit of equation 6 suggests, erroneously, that a small fraction of L cone pigment is present at the fovea of the person with only M cones. (Top right) With the correction, the spectra are shifted toward shorter wavelengths and the fit is optimised ($R^2$=0.99) with M-cone, L-cone and meta-iodopsin III densities of 0.170, 0.000, 0.018 respectively. (Bottom row) Analogous data for the observer with only L-cones at the fovea. The scatter correction shifts the data to shorter wavelengths and boosts the total density difference. In this case (Bottom right), the fit is optimised ($R^2$=0.93) with M-cone, L-cone and meta-iodopsin III densities of 0.000, 0.285, 0.023 respectively. Error bars describe the magnitude of the standard error.*

Implementation of the scatter correction shifted the density difference spectra to shorter wavelengths by ~10nm and increased the peak OD by ~30% (Figure S4). The effect of this correction was to bring the post bleach density difference spectra into close alignment with established human M and L cone visual pigment spectra[3]. The fit was optimised by correcting for a small fraction of meta-iodopsin III. However, the meta-iodopsin III correction was only needed to accommodate a modest mismatch between the cone density difference data and the msp reference spectra. An alternative explanation for the mismatch, and apparent presence of meta-iodopsin III for cones, relates to the msp spectra used[3] which have been reported to overestimate absorption particularly at shorter wavelengths[7].

In the temporal retina, the scatter correction also shifted the spectra to shorter wavelengths but despite this a significant fraction (~30%) of metarhodopsin III was still required to bring the msp spectrum for the rod pigment, rhodopsin, into register with the measured density difference spectra. The effect of the scatter correction on the density difference measured in the temporal retina is exemplified in the bottom row of Figure S3.

The successful recovery of cone visual pigment spectra *in vivo* in individuals with known colour vision deficiencies and the identification of both rhodopsin and metarhodopsin III in the temporal retina, in accordance with earlier reports[6], provided reassurance that our approach to IRD worked.

**Characteristics of participants with AMD**: Five people with age-related macular degeneration participated in this study. The characteristics of these participants is summarised in Table 1. All grading was undertaken by one of the authors (AW), an experienced optometrist. Fundus photographs were graded according to the Beckmann grading scale. Topcon 3D OCT images were reviewed to improve the description of retinal status.

**Table 1.** Characteristics of participants with AMD. The Beckman grading scale was used to classify retinal appearance on the basis of colour fundus photography[8]. Additional information about the status of the study eye was derived from OCT imagery.

| Patient ID | Age | M/F | Study Eye | | | | | Second Eye | | Smoking status |
|---|---|---|---|---|---|---|---|---|---|---|
| | | | RE/LE | Beckman grade | VA | OCT observations[1] | Retinal thickness[2] | Beckman grade | VA | |
| 1 | 77 | M | LE | iAMD | 0.22 | Drusen and Ez (ellipsoid zone) disruption in central subfield only. | 243μm | iAMD | 0.26 | Previous 14 pack years |
| 2 | 71 | F | RE | iAMD | 0.18 | Drusen in all subfields, increased size & confluence in central and parafoveal subfields. | ~168μm | iAMD | 0.38 | Previous 10 pack years |
| 3 | 68 | F | LE | iAMD | 0.24 | Drusen largely confined to central subfield. | 241μm | iAMD | 0.02 | Never |
| 4 | 68 | F | LE | iAMD | -0.20 | Drusen largely confined to central and parafoveal subfields, Ez disruption in central subfield. | 265μm | iAMD | -0.20 | Previous <5 pack years |
| 5 | 85 | M | LE | iAMD | -0.06 | Drusen and Ez disruption largely confined to nasal parafoveal subfield. | 259μm | iAMD | -0.02 | Previous 5 pack years |

1. Locations described on basis of ETDRS grid sub-fields.
2.ETDRS central sub-field thickness.

**Image processing:** Images from the recording session were processed in a data reduction pipeline (DRP), implemented using Interactive Data Language (IDL), that included the following steps: 1) CCD bias and stray light within the densitometer system, determined at the start of each session by obtaining images in complete darkness, was subtracted from each frame. 2) Following image registration, the signal from an interleaved frame that contained residual light from the blue fixation cross only and infrared tracking LEDs was also subtracted. 3) To account for any temporal instability in the output of the LEDs, images were scaled continuously according to the output of the integrating sphere. 4) the images were flat fielded and converted into absolute reflectance values by dividing the images by those obtained from a model eye with a retina made of PTFE (reflectance ~ 100% across all wavelengths). 5) Finally, images were corrected for back scatter using the Mie scattering function

$$\mu_s'(\lambda) = a' \left( f_{\text{Ray}} \left( \frac{\lambda}{475\ (\text{nm})} \right)^{-4} + \left(1 - f_{\text{Ray}}\right) \left( \frac{\lambda}{475\ (\text{nm})} \right)^{-b_{\text{Mie}}} \right) \tag{1}$$

where $\mu_s'$ is the scattering coefficient, $a'$ is the value of $\mu_s'$ at 475nm i.e. a scaling factor, 1-$f_{Ray}$ is the fraction of Mie scattering and $b_{Mie}$ is scattering power[1]. Here $a'$ was set to be the mean signal measured at the center of the bar mask at 475nm multiplied by 1.16 to account for light lost in the bar mask. A second scatter correction, which varied topographically according to the distribution of the RNFL (assessed on the basis of OCT imagery), was also applied. The amplitude of this correction brought the spectral profile obtained from the nasal retina into register with that from the temporal retina in healthy controls and was subsequently fixed. The decision to standardise the amplitude of this second correction across participants was based on the observation that the normal variation in RNFL thickness is relatively modest (100.9 SD11μm)[9]. For both scatter corrections the constants $f_{Ray}$ and $b_{Mie}$ were set to 0.409 and 0.702 respectively[1].

The resultant DA and BR image stacks provided high fidelity, scatter free, measurements of retinal reflectance in 4 dimensions (x, y, λ, t).

**Spectral and temporal fitting.** To determine post bleach rod and cone density difference, the processed DA image sequence was temporally averaged ($DA_{x,y,\lambda}$) and the density difference spectra at each point in time ($DD_{x,y,\lambda,t}$) calculated as

$$DD_{x,y,\lambda,t} = \log10\ (BR_{x,y,\lambda,t} / DA_{x,y,\lambda}). \tag{2}$$

Subsequently, rod and cone spectra were fitted to the post bleach density difference spectra using a least squares approach to recover the time dependent density difference attributable to rods and cones. Firstly, to stabilise the fitting across the retina, M and L cone spectra were only fitted independently at the fovea, where there are no rods

$$DD_{fovea,\lambda} = DD_{Mcone} + DD_{Lcone} \tag{3}$$

where $DD_{Mcone}$ and $DD_{Lcone}$ were the scaled spectral profiles for the M and L cone visual pigments as determined by Dartnall et al, (1983) [3] and interpolated to match the wavelengths of the individual LEDs. This produced an M to L cone ratio for each individual and resulted in a cone density difference spectral profile which was assumed to remain stable across the retina[10]

$$DD_{cone} = DD_{Mcone} + DD_{Lcone}. \tag{4}$$

Subsequently, rod and cone density difference data were determined topographically across the retina on the basis of the density difference spectra

$$DD_{x,y,\lambda,t} = DD_{rod,t} + DD_{cone,t} \tag{5}$$

where $DD_{rod}$ was the rod spectral profile based on that proposed by Dartnall et al, (1983) which varied in amplitude as a function of time.

In some instances when we sought to quantify the effects of meta III, the density difference data at the fovea were fitted using

$$DD_{fovea,\lambda,t} = DD_{Mcone,t} + DD_{Lcone,t} - OD_{meta,t} \tag{6}$$

where $OD_{meta}$ was the spectral profile for Metarhodopsin III determined by Frederiksen et al, (2016)[11]. That is, we assumed that the absorption characteristics of the cone bleaching photoproduct known as meta-iodopsin III was identical to that of the rod bleaching photoproduct metarhodopsin III. We made

this assumption because there is currently no good data on the absorption characteristics of meta-iodopsin III. In other instances when we wished to quantify the effect of meta III topographically we used,

$$DD_{x,y,\lambda,t} = DD_{rod,x,y,t} + DD_{cone,x,y,t} - OD_{meta,x,y,t} \quad (7)$$

and in the person with no measurable cone contribution to the post bleach density difference spectra,

$$DD_{x,y,\lambda,t} = DD_{rod,x,y,t} - OD_{meta,x,y,t}. \quad (8)$$

To characterise the temporal characteristics of the recovery two expressions were fitted to the data (Fig. 3): either a simple exponential recovery,

$$P(t) = OD.(1\text{-}\exp(-t/\tau) \quad (9)$$

where $P$ is the amount of visual pigment present at time $t$, $OD$ is the final optical density and $\tau$ is the time constant of recovery, or the rate-limited expression developed by Mahroo and Lamb (2004) i.e. their equation (A6)

$$NP(t) = 1 - K_m W\left\{\frac{B}{K_m}\exp\left(\frac{B}{K_m}\right)\exp\left(-\frac{1+K_m}{K_m}vt\right)\right\} \quad (10)$$

where $NP$ is the fraction of visual pigment, $W$ denotes the 'lambert W function', defined as the function that satisfies $W(x)e^{W(x)}=x$, and where $B$ is the initial size of the bleach which was assumed to be 0.98 for rods and 0.95 for cones, $K_m$ is the Michaelis constant and was assumed to be 0.2 and $v$ is the initial slope of the normalized recovery. This function was rescaled to determine absolute optical density values

$$P(t) = OD.NP(t). \quad (11)$$

To characterise visual pigment recovery rates topographically, equations 10 and 11 were fitted to the data on a pixel by pixel basis. The resultant data were summarised using topographical heat maps that describe rod and cone visual pigment synthesis rates across the central retina (e.g. Fig. 5).

A single PC was used to implement the Java based pupil fitting and Lab View based robotics control of the optical system which happened in 'real time'. The Interactive Data Language (IDL) based imaging processing ran in the background on the same PC and typically completing the processing within 10 seconds of the end of each experiment. Data generated by the IDL routines was transferred to Microsoft Excel for Mac (V 16.13.1) and Matlab (R2016b) to produce the spectral and temporal profiles shown here. The heatmap images were generated from the image data produced by the IDL routines manipulated using the 'fire' look up table and calibration bar in Image J (2.0.0-rc-59/1.5k).

**System calibrations:** An ILT1700 research radiometer fitted with a SED033 photopic detector was used to determine the photopic output of the IRD and data from Wyszecki and Stiles[12] were used to convert this into scotopic units. The spectral characteristics of the IRD's LEDs, the absorption characteristics of the 'visual pigment' like filter used in the model eye and the model eye's 'retinal' reflectance was determined using an Ocean Optics Flame-S-VIS-NIR-ES spectrometer. Absolute reflectance measurements from the eye were determined using a diffuse 'white' PTFE surface supplied by Lab Sphere as a reference standard.

**References:**

1. Jacques SL. Optical properties of biological tissues: a review. *Physics in Medicine and Biology.* 2013;58(11):R37-R61.
2. Mahroo OAR, Lamb TD. Recovery of the human photopic electroretinogram after bleaching exposures: estimation of pigment regeneration kinetics. *Journal of Physiology-London.* 2004;554(2):417-437.
3. Dartnall HJA, Bowmaker JK, Mollon JD. Human visual pigments - microspectrophotometric results from the eyes of 7 persons. *Proceedings of the Royal Society Series B-Biological Sciences.* 1983;220(1218):115-130.

4. Kolesnikov AV, Golobokova EY, Govardovskii VI. The identity of metarhodopsin III. *Visual Neuroscience.* 2003;20(3):249-265.
5. Ripps H, Weale RA. Flash bleaching of rhodopsin in human retina. *Journal of Physiology-London.* 1969;200(1):151-159.
6. Ripps H, Weale RA. Rhodopsin regeneration in man. *Nature.* 1969;222(5195):775-777.
7. Stockman A, Sharpe LT. The spectral sensitivities of the middle- and long-wavelength-sensitive cones derived from measurements in observers of known genotype. *Vision Research.* 2000;40(13):1711-1737.
8. Ferris FL, Wilkinson CP, Bird A, et al. Clinical classification of age-related macular degeneration. *Ophthalmology.* 2013;120(4):844-851
9. Budenz DL, Anderson DR, Varma R, et al. Determinants of normal retinal nerve fiber layer thickness measured by stratus OCT. *Ophthalmology.* 2007;114(6):1046-1052.
10. Nerger JL, Cicerone CM. The ratio of L-cones to M-cones in the human parafoveal retina. *Vision Research.* 1992;32(5):879-888.
11. Frederiksen R, Nymark S, Kolesnikov AV, et al. Rhodopsin kinase and arrestin binding control the decay of photoactivated rhodopsin and dark adaptation of mouse rods. *Journal of General Physiology.* 2016;148(1):1-11.
12. Wyszecki G, Stiles WS. *Color Science: Concepts and Methods, Quantitative Data and Formulae.* 2nd ed. New York: John Wiley and Sons; 1982.
